# Genome Sequences of Ambystoma Tigrinum Virus Recovered during a Mass Die-off of Western Tiger Salamanders in Alberta, Canada

**DOI:** 10.1128/MRA.00265-19

**Published:** 2019-07-18

**Authors:** Oliver Lung, Michelle Nebroski, Shivani Gupta, Cameron Goater

**Affiliations:** a National Centre for Foreign Animal Disease, Canadian Food Inspection Agency, Winnipeg, MB, Canada; b Department of Biological Sciences, University of Manitoba, Winnipeg, MB, Canada; c Department of Biological Sciences, University of Lethbridge, Lethbridge, AB, Canada; KU Leuven

## Abstract

Complete genome sequences of six Ambystoma tigrinum viruses (ATV) were determined directly from tail clips of western tiger salamanders (Ambystoma mavortium) from 2013 (high-mortality year) and 2014 (low-mortality year) in Alberta, Canada. The genome lengths ranged from 106,258 to 106,915 bp and contained 108 open reading frames encoding predicted proteins larger than 50 amino acids.

## ANNOUNCEMENT

Ambystoma tigrinum virus (ATV), a virus with a double-stranded DNA (dsDNA) genome of ∼106 kb (1), and other emerging viruses of the genus Ranavirus and family Iridoviridae affect a wide range of amphibians, reptiles, and fish (2, 3). To allow identification of candidate pathogenicity markers, DNA was extracted from the tail clips of tiger salamanders collected from Livingston Lake in Southern Alberta, Canada, during massive die-offs in 2013 with close to 100% mortality and during a year with almost no observed mortality, 2014. Archived DNA from samples positive for ATV by insulated isothermal PCR (iiPCR), which utilizes a temperature gradient instead of thermal cycling (4), was used to determine the full viral genome in five specimens collected in 2013 and one specimen collected in 2014 (Table 1). DNA was quantified using the Qubit 2.0 fluorometer (Thermo Fisher) and normalized to a 5 ng/μl concentration with resuspension buffer (RSB) from the TruSeq Nano DNA kit for NeoPrep (Illumina) in a final volume of 15 μl. DNA was sheared to ∼550-bp fragments using an M220 ultrasonicator (Covaris) and processed on an Illumina NeoPrep instrument for sequence library preparation. Normalized sequencing library pools were sequenced on an Illumina MiSeq instrument using a V3 flow cell and a 600-cycle reagent cartridge (Table 1).

**TABLE 1:** Summary of the assembly and annotation of ATVs^*a*^

Sample	Total no. of reads	Length^*b*^ (bp)	Coverage^*f*^ (×)	% identity to closest reference^*g*^	MMP^*c*^ size (bp)	NTHP^*d*^ size (bp)
2013-ATV-04	4,990,944	106,258	11.1040	99.21	948	795
2013-ATV-12	2,438,202	106,652	67.1694	99.21	972	831
2013-LL1	4,577,472	106,578	86.0044	99.18	996	831
2013-LL2	8,196,768	106,730	88.7567	99.18	996	831
2013-LL3	3,406,280	106,629	63.5509	99.18	972	831
2014-ATV-322^*e*^	1,741,656	106,915	115.881	99.21	1,020	993

a All samples isolated from 2013 were from a high-mortality year with close to 100% mortality, while the sample from 2014 was from a low-mortality year with almost no observed mortality. All samples had a GC content of 53.8% and had 108 ORFs encoding predicted proteins larger than 50 amino acids.

b After correction of assemblies in Geneious.

c MMP, putative myristoylated membrane protein.

d NTHP, neurofilament triplet H1-like protein.

e Enriched using the ViroCap method (7) prior to sequencing due to low viral titer.

f Determined using SPAdes.

g The top BLAST match for all samples was Ambystoma tigrinum stebbensi virus (GenBank accession number AY150217).

Following sequencing, the full viral genomes were generated by trimming raw reads using Trimmomatic (5) and then using SPAdes (v.3.9.1) (6) for *de novo* assembly. For samples 2013-ATV-04, 2013-LL1, and 2013-LL3, the trimming parameters were SLIDINGWINDOW:4:20 and MINLEN:75, and for samples 2013-ATV-12 and 2013-LL2, they were HEADCROP:15, SLIDINGWINDOW:4:20, TRAILING:20, and MINLEN:75. The number of untrimmed ATV reads in the sixth DNA sample (2014-ATV-322) was 495 (from a total of 2,711,354 reads), which resulted in only a partial genome using SPAdes in metagenomics mode (metaSPAdes). Therefore, the sample was resequenced using probe capture enrichment (7). Resequencing was performed on an Illumina MiSeq instrument using a V2 flow cell and a 300-cycle reagent cartridge (Table 1). The complete genome was assembled after filtering the reads using drVM (8) and then running the resulting Ranavirus reads through SPAdes in careful mode.

Raw sequencing reads from each sample were mapped back to the assembled genomes in Geneious (v.3.11.4) (9) and manually corrected for errors in the assemblies. Prior to read mapping, the assembled genomes were circularized in Geneious to observe that the reads on the ends wrapped around the genome, therefore confirming their completeness. The complete genomes were searched for open reading frames (ORFs), using the Geneious ORFfinder, with a minimum length of 150 nucleotides. Coding sequences (CDS) from 15 complete ATV genomes imported from NCBI (23 June 2017) were transferred to the six samples using the Geneious Live Annotate and Predict tool with a 65% nucleotide identity threshold. An ORF and a CDS at the same position were considered a predicted protein. The minimum ORF length was then lowered to 48 nucleotides to find the remaining smaller CDS. GeneMarkS (10) and a local version of NCBI ORFfinder (11) were used to confirm the ORFs annotated in Geneious. Amino acid sequences found by the NCBI ORFfinder were run through the blastp command in DIAMOND (12) using a database of viral proteins downloaded from the NCBI (4 May 2018).

Two differences observed between the high-mortality- and low-mortality-year samples include variations in the sizes of the ORFs for the putative myristoylated membrane protein (MMP) (corresponding to nucleotide position 11 to 1,030 of genome 2014-ATV-322 [GenBank accession number MK580536]) and the small neurofilament triplet H1-like protein (NTHP) (corresponding to nucleotide position 83798 to 84790 of genome 2014-ATV-322 [MK580536]) (Table 1), with the ORFs for the low-mortality-year samples being larger than those from the high-mortality-year samples. These differences may impact pathogenicity; however, further experimentation is required to confirm this.

### Data availability.

Nucleotide sequences, including annotations and raw sequencing reads, were deposited in GenBank and the Sequence Read Archive (SRA), National Center for Biotechnology Information, with the following GenBank and SRA accession numbers, respectively: MK580531 and SAMN11127530 (2013-ATV-04); MK580532 and SAMN11127531 (2013-ATV-12); MK580533 and SAMN11127532 (2013-LL1); MK580534 and SAMN11127533 (2013-LL2); MK580535 and SAMN11127534 (2013-LL3); and MK580536 and SAMN11127535 (2014-ATV-322). The versions described in this paper for MK580531, MK580532, MK580533, and MK580535 are the second versions; all other versions described are the first versions.
